# Systematic review and network meta-analysis of interventions for fibromyalgia: a protocol

**DOI:** 10.1186/2046-4053-2-18

**Published:** 2013-03-13

**Authors:** Jason W Busse, Shanil Ebrahim, Gaelan Connell, Eric A Coomes, Paul Bruno, Keshena Malik, David Torrance, Trung Ngo, Karin Kirmayr, Daniel Avrahami, John J Riva, Peter Struijs, David Brunarski, Stephen J Burnie, Frances LeBlanc, Ivan A Steenstra, Quenby Mahood, Kristian Thorlund, Victor M Montori, Vishalini Sivarajah, Paul Alexander, Milosz Jankowski, Wiktoria Lesniak, Markus Faulhaber, Małgorzata M Bała, Stefan Schandelmaier, Gordon H Guyatt

**Affiliations:** 1 Department of Anesthesia, McMaster University, 1200 Main Street West, Hamilton, Ontario, L8S 4K1, Canada; 2 Department of Clinical Epidemiology and Biostatistics, McMaster University, 1200 Main Street West, Hamilton, Ontario, L8S 4K1, Canada; 3 Canadian Memorial Chiropractic College, 6100 Leslie Street, Toronto, Ontario, M2H 3J1, Canada; 4 Faculty of Medicine, University of Toronto, 1 Kings College Circle, Toronto, Ontario, M5S 1A8, Canada; 5 Faculty of Kinesiology and Health Studies, University of Regina, 3737 Wascana Parkway, Regina, Saskatchewan, S4S 0A2, Canada; 6 School of Rehabilitation Sciences, McMaster University, 1400 Main Street West, Hamilton, Ontario, L8S 1C7, Canada; 7 German Hospital, Av. Pueyrredón 1640 (C1118 AAT), Buenos Aires, Argentina; 8 Department of Family Medicine, McMaster University, McMaster Innovation Park, 175 Longwood Road South, Hamilton, Ontario, L8P 0A1, Canada; 9 Department of Orthopaedic Surgery, Academic Medical Center, Meibergdreef 9, 1105, Amsterdam-Zuidoost, the Netherlands; 10 Ontario Chiropractic Association, 20 Victoria Street, Suite 200, Toronto, Ontario, M5C 2N8, Canada; 11 The Institute for Work & Health, 481 University Avenue, Toronto, Ontario, M5G 2E9, Canada; 12 Dalla Lana School of Public Health, University of Toronto, 6th floor, 155 College Street, Toronto, Ontario, M5T 3M7, Canada; 13 Stanford Prevention Research Center, Stanford University School of Medicine, 1265 Welch Road, Stanford, CA, 94305-5411, USA; 14 Knowledge and Evaluation Research Unit, Divisions of Endocrinology and Diabetes and Health Care and Policy Research, Mayo Clinic, 200 First Street SW, Rochester, MN, 55905, USA; 15 Faculty of Health Sciences, McMaster University, 1280 Main Street West, Hamilton, Ontario, L8S 4K1, Canada; 16 2nd Department of Internal Medicine, Jagiellonian University Medical College, ul Skawinska 8, 31-066, Kraków, Poland; 17 Polish Institute of Evidence Based Medicine, ul. Krakowska 41, 31-066, Kraków, Poland; 18 Academy of Swiss Insurance Medicine, University Hospital Basel, Schanzenstrasse 55, 4031, Basel, Switzerland

**Keywords:** Fibromyalgia, Systematic review, Network meta-analysis, Multiple treatment comparison, Randomized controlled trials

## Abstract

**Background:**

Fibromyalgia is associated with substantial socioeconomic loss and, despite considerable research including numerous randomized controlled trials (RCTs) and systematic reviews, there exists uncertainty regarding what treatments are effective. No review has evaluated all interventional studies for fibromyalgia, which limits attempts to make inferences regarding the relative effectiveness of treatments.

**Methods/design:**

We will conduct a network meta-analysis of all RCTs evaluating therapies for fibromyalgia to determine which therapies show evidence of effectiveness, and the relative effectiveness of these treatments. We will acquire eligible studies through a systematic search of CINAHL, EMBASE, MEDLINE, AMED, HealthSTAR, PsychINFO, PapersFirst, ProceedingsFirst, and the Cochrane Central Registry of Controlled Trials. Eligible studies will randomly allocate patients presenting with fibromyalgia or a related condition to an intervention or a control. Teams of reviewers will, independently and in duplicate, screen titles and abstracts and complete full text reviews to determine eligibility, and subsequently perform data abstraction and assess risk of bias of eligible trials. We will conduct meta-analyses to establish the effect of all reported therapies on patient-important outcomes when possible. To assess relative effects of treatments, we will construct a random effects model within the Bayesian framework using Markov chain Monte Carlo methods.

**Discussion:**

Our review will be the first to evaluate all treatments for fibromyalgia, provide relative effectiveness of treatments, and prioritize patient-important outcomes with a focus on functional gains. Our review will facilitate evidence-based management of patients with fibromyalgia, identify key areas for future research, and provide a framework for conducting large systematic reviews involving indirect comparisons.

## Background

Fibromyalgia is a syndrome characterized by chronic widespread pain and excessive tenderness at 11 of 18 specific muscle-tendon sites, for which no clear cause can be found [1]. Approximately 2% of the general population in the United States suffers from fibromyalgia, with women affected ten times more often than men [2]. Similar prevalence rates have been reported in Canada (3.3%), Brazil (4.4%) and Western European countries, including Germany (3.2%), Spain (2.4%), Italy (2.2%), Sweden (2.5%), France (1.4%), Italy (3.7%) and Portugal (3.6%) [3-8]. Treatment directed towards fibromyalgia is highly variable and long-term prospective observational studies have found that patient outcomes, even in specialized rheumatology clinics, are typically poor [9,10].

Poor treatment outcomes have led to frustration among patients and their clinicians, and contributed to the impact of fibromyalgia on disability [11-13]. Observational studies have found that 20% to 50% of persons with fibromyalgia report that they are unable to work or that they can work only a few days per month, and 27% to 55% receive disability or social security payments. Of those that do work, 36% experience an average of two or more absences from work per month [11-13].

Physicians typically find fibromyalgia difficult to manage and patients with fibromyalgia are likely to be dissatisfied with treatment [14]. A recent survey of 1,200 primary care physicians in the United States (33% response rate) found that only 14% of respondents indicated very good or excellent satisfaction with managing patients with fibromyalgia and other medically unexplained symptoms [15], and a study of 400 British general practitioners (75% response rate) found that only 44% of respondents felt there were effective treatment options available for this population [16].

There have been no less than 38 systematic reviews addressing therapies for fibromyalgia [17-54]; however, the large majority of reviews to date have explored therapies in isolation or looked at a subgroup of treatments. There have been two network meta-analyses we are aware of that explored multiple therapies for fibromyalgia [51,52]. The first [52] restricted comparisons to pharmacological treatments only and used the Jadad scale to assess study quality [55], which has been criticized as overly simplistic and placing too much emphasis on blinding [56]. The second [51] excluded complementary and alternative medicine approaches, had no assessment of study quality, and summarized continuous data using the standardized mean difference, which is vulnerable to differential variability in populations enrolled and adds challenges in interpreting the magnitude and importance of treatment effects [57,58]. No review has looked at all interventional studies for fibromyalgia, which limits attempts to make inferences regarding the relative effectiveness of available treatments. Further, none used the GRADE framework to establish confidence in pooled estimates of treatment effect [59].

We will explore all therapies for fibromyalgia that have been tested in randomized controlled trials (RCTs) and use Bayesian mixed treatment comparison methods (adjusted indirect comparisons) to complement the direct comparisons of the relative effects of competing interventions in RCTs [60,61].

## Methods/design

### Protocol and registration

Our protocol is registered on PROSPERO (CRD42012003291), http://www.crd.york.ac.uk/PROSPERO.

Our paper conforms to the PRISMA guidelines for reporting systematic reviews [62].

### Eligibility criteria

Trials eligible for our review will (1) have enrolled adult patients (≥18 years of age) presenting with fibromyalgia or a related condition (for example, myofascial pain syndrome, fibrositis, fibromyositis, muscular rheumatism, chronic generalized pain syndrome), and (2) have randomized patients to an intervention or a control arm.

### Information sources and search

We will identify relevant RCTs, in any language, by a systematic search of CINAHL, EMBASE, MEDLINE, AMED, HealthSTAR, PsycINFO, PapersFirst, ProceedingsFirst, and the Cochrane Central Registry of Controlled Trials, from inception of the database, with relevant MeSH headings. An experienced librarian (QM) developed a sensitive search strategy for each individual database (see Additional file 1). We will scan the bibliographies of all retrieved trials and other relevant publications, including reviews and meta-analyses, for additional relevant articles.

### Study selection

Twenty reviewers with experience in health research methodology will work in pairs to screen, independently and in duplicate, titles and available abstracts of identified citations and acquire the full text publication of any article that either reviewer judge as potentially eligible. The same reviewer teams will independently apply eligibility criteria to the full text of potentially eligible trials. Reviewers will resolve disagreements by consensus or, if a discrepancy remains, through discussion with one of two arbitrators (JWB or GHG).

### Data collection process and data items

Using standardized forms (see Additional file 2) and a detailed instruction manual that will be used to inform specific tailoring of an online data abstraction program (DistillerSR), ten teams of reviewers will extract data independently and in duplicate from each eligible study. To ensure consistency across reviewers, we will conduct calibration exercises before starting the review. Data abstracted will include demographic information, methodology, intervention details, and all reported patient-important outcomes. Reviewers will resolve disagreements by discussion, and one of two arbitrators (JWB or GHG) will adjudicate unresolved disagreements. We will contact study authors to resolve any uncertainties.

Two reviewers (GC, EAC) will independently extract details on interventions and outcomes from all RCTs in order to classify them into common intervention categories. The abstractors will develop the categories independently and then achieve consensus through discussion. Outcomes will be classified into domains, based on the guidelines published by the Initiative on Methods, Measurement, and Pain Assessment in Clinical Trials (IMMPACT) [63-70]. These consist of nine domains: pain; physical and emotional functioning (including Quality of Life); participant rating of improvement and satisfaction with treatment; adverse events; participant disposition (for example, adherence to the treatment regime and reasons for premature withdrawal from the trial); role functioning (that is, work and educational activities, social and recreational activities, home and family care); interpersonal functioning (that is, interpersonal relationships, sexual activities); sleep; and fatigue.

### Risk of bias in individual studies

Reviewers will assess risk of bias within each study with a modified Cochrane risk of bias instrument which assesses the following key domains: sequence generation; allocation concealment; blinding of participants, healthcare professionals, outcome assessors, data collectors, and data analysts; incomplete outcome data; selective outcome reporting; and other sources of bias. Reviewers will input response options of ‘definitely yes’, ‘probably yes’, ‘probably no’, and ‘definitely no’ for each of the domains, with ‘definitely yes’ and ‘probably yes’ ultimately assigned low risk of bias and ‘definitely no’ and ‘probably no’ assigned high risk of bias [71]. Reviewers will resolve disagreements by discussion, and one of two arbitrators (JWB or GHG) will adjudicate unresolved disagreements.

### Direct comparisons meta-analyses

To pool outcome data for trials that compare the same intervention with the same comparator, we will use random effects meta-analyses, which are conservative in that they consider both within and among study differences in calculating the error term used in the analysis [11]. We will pool cross-over trials with parallel design RCTs, using methods outlined in the Cochrane handbook to derive effect estimates [72]. Specifically, we will perform a paired *t*-test for each cross-over trial if either of the following are available: 1) the individual participant data; 2) the mean and SD (or standard error) of the participant-specific differences between the intervention and control measurements; 3) the mean difference and one of the following: (i) a t-statistic from a paired *t*-test, (ii) a p-value from a paired *t*-test, or (iii) a confidence interval from a paired analysis; or 4) a graph of measurements of the intervention arm and control arm from which we can extract individual data values (pending that the matched measurements for each individual can be identified) [72]. If these data are not available, we will approximate paired analyses by first calculating the mean difference for the paired analysis (MD = M_E_ - M_C_) and the standard error of the mean difference: $\mathrm{SE}\left(\mathrm{MD}\right)=\frac{SD_{\mathrm{diff}}}{\sqrt{N}}$, where N represents the number of participants in the trial, and SD_diff_ represents the standard deviation of within-participant differences between the intervention and control measurements [72]. If the standard error or standard deviation of within-participant differences is not available, we will impute the standard deviation using methods outlined in the Cochrane Handbook [73].

### Ensuring interpretable results from pooled estimates of effect

We will use a number of approaches to facilitate interpretable results from our meta-analyses. For trials that report dichotomous outcomes, we will calculate the odds ratio (OR) to inform relative effectiveness. We will acquire estimates of baseline risk from observational studies or, if not available, from the median of the control group from eligible RCTs.

When pooling across trials that report continuous outcomes using the same instrument, we will calculate the weighted mean difference (WMD), which maintains the original unit of measurement and represents the average difference between groups [74]. Once the WMD has been calculated, we will contextualize this value by noting the corresponding minimally important difference (MID) - the smallest change in instrument score that patients perceive is important. We will prioritize use of anchor-based MIDs when available, and calculate distribution-based MIDs when they are not.

Contextualizing the WMD through the MID can be misleading because clinicians may interpret a WMD less than the MID as suggesting that no patient obtains an important benefit, which is not accurate. Therefore, we will generate an estimate of the proportion of patients who have benefited by applying the MID to individual studies, estimating the proportions who benefit in each study, and then aggregate the results in order to provide a summary estimate of the proportion of patients who benefit from treatment across all studies. Details of the methods by which we will conduct this analysis are presented immediately below in our discussion of situations in which investigators have used different instruments to measure the same construct.

For trials that use different continuous outcome measures that address the same underlying construct, one cannot calculate a weighted mean difference, and we will therefore calculate a measure of effect called the standardized mean difference (SMD) or ‘effect size’. This involves dividing the difference between the intervention and control means in each trial (that is, the mean difference) by the estimated between-person standard deviation (SD) for that trial. The SMD expresses the intervention effect in SD units rather than the original units of measurement, with the value of a SMD depending on both the size of the effect (the difference between means) and the SD of the outcomes (the inherent variability among participants).

This common approach to pooling continuous outcome data is often problematic. If the heterogeneity of patients is different across studies, the SD will vary across studies. Therefore, given the same true difference in outcome between intervention and control groups, trials with more heterogeneous patients will show apparently - but spuriously - smaller effects than trials enrolling less heterogeneous patients. Furthermore, interpretation of the magnitude of effect when represented as SD units is not intuitive.

In order to address these issues, we will contextualize the SMD value through MID units, which are not vulnerable to the distortions that varying heterogeneity of populations can create and are more interpretable to both clinicians and patients [58,75]. For outcome measures that have an established anchor-based MID we will use this measure to convert the summary effect into OR. We will complement this presentation by either converting the summary effect into natural units of a widely accepted instrument used to measure changes in the domain of interest or, if such an instrument is not available, we will substitute the MID for the SD (denominator) in the SMD equation, which will result in more readily interpretable MID units instead of SD units [58]. Finally, we will, as for SMD, provide a summary estimate of the proportion of patients who benefit from treatment across all studies

We illustrate this approach with the following example. We will first describe how we will summarize the outcome in MID units. Assume that a trial reports a mean difference (MD) on a continuous outcome measure “X”, and assume that an anchor-based MID for instrument X, MID_X_, has been established. The estimated MD is a random variable. If we standardize this random variable by dividing it by the MID_X_, we get a new random variable, MD/MID_X_. We know from basic probability theory that because MID_X_ is a constant, the variance of MD/MID_X_ is given by:

$$\mathrm{Var}\left(\frac{\mathrm{MD}}{\mathrm{MI}D_{X}^{2}}\right)=\frac{\mathrm{Var}\left(\mathrm{MD}\right)}{\mathrm{MI}D_{X}^{2}}$$

That is, the variance of the mean difference divided by the square of the MID. Further, the standard error of MD/MID_X_ is given by:

$$\mathrm{SE}\left(\frac{\mathrm{MD}}{\mathrm{MI}D_{X}^{2}}\right)=\sqrt{\frac{\mathrm{Var}\left(\mathrm{MD}\right)}{\mathrm{MI}D_{X}^{2}}}=\frac{\mathrm{SE}\left(\mathrm{SD}\right)}{\mathrm{MI}D_{X}}$$

Consider a meta-analysis that included k trials. The first j trials use disease-specific instrument A, and the last k-j trials use disease-specific instrument B. Let MD_i_ denote the mean difference observed in trial i, let MID_A_ denote the MID established for instrument A, and let MID_B_ denote the MID established for instrument B. Further, let m_i_ denote the MID standardized effect for trial i. To pool results across trials using MIDs we must first estimate the m_i_ and its associated variance for all trials. For i = 1, …, j we have:

$$m_{i}=\frac{MD_{i}}{\mathrm{MI}D_{A}}\;\mathrm{and}\;\mathrm{Var}\left(m_{i}\right)=\frac{\mathrm{Var}\left(MD_{i}\right)}{\mathrm{MI}D_{A}^{2}}$$

and for i = j + 1, …, k we have:

$$m_{i}=\frac{MD_{i}}{\mathrm{MI}D_{B}}\;\mathrm{and}\;\mathrm{Var}\left(m_{i}\right)=\frac{\mathrm{Var}\left(MD_{i}\right)}{\mathrm{MI}D_{B}^{2}}$$

By defining the trial weights as w_i_ = Var(m_i_)^-1^, we can use the fixed-effect model inverse variance method to pool the MID-standardized mean differences using the formula:

$$\hat{m}=\left(\sum_{i=1}^{k}w_{i}\cdot m_{i}\right)/\left(\sum_{i=1}^{k}w_{i}\right)$$

Where $\hat{m}$ denotes the pooled MID-standardized mean difference. The standard error of $\hat{m}$ can be calculated using the formula:

$$\mathrm{se}\left(\hat{m}\right)=1/\left(\sum_{i=1}^{k}w_{i}\right)$$

The associated confidence intervals can subsequently be derived. MID-standardized mean differences can also be combined in a random-effects model using weights w_i_ = (Var(m_i_) + τ^2^)^-1^, where τ^2^ denotes the between-trial variance.

This presentation does not address the risk that clinicians may interpret all mean effects below the MID as unimportant, and presume important benefit for all patients when mean effects exceeds the MID. We will address this issue by assuming normal distributions of data and then calculating the proportions of participants in the intervention and control groups in each study that demonstrated an improvement greater than the MID [76]. The results are then pooled across studies. If we only have post-test data (rather than magnitude of change), we will apply this approach if evidence exists regarding meaningful thresholds. For instance, if one knows that people with scores of less than 8 on the Hamilton rating scale for depression (HAM-D) are considered to be not depressed, one could examine the proportion of individuals below that threshold.

If such meaningful thresholds do not exist, we will use post-test data and assume that the minimally important change within an individual corresponds, on average, to the minimally important difference between individuals. Making this assumption, one can calculate the difference in the proportion who benefit in intervention and control. To do this, we will take the mean value in the control group plus one MID unit, and calculate the proportion of patients in each group above that threshold.

If an anchor-based MID has not been established for all instruments, we will assume a meta-analysis control group probability (*p*_*C*_) and use the SMD to calculate the OR. Specifically, we will construct a conceptual meta-analysis control group with mean *μ*_*C*_, standard deviation *σ*_*C*_, and group size *n*_*C*_, and a conceptual meta-analysis intervention group with mean *μ*_*E*_, standard deviation *σ*_*E*_, and group size *n*_*E*_ such that the SMD can be represented as *SMD = μ*_*E*_ - *μ*_*C*_ and *σ*_*E*_ *= σ*_*C*_ *= 1*. We will set *μ*_*C*_ *= 0* and *σ*_*C*_ *= 1*, and our threshold (*T)* will be equal to *Φ*^*-1*^*(p*_*C*_*),* where *Φ*^*-1*^ is the inverse standard normal cumulative distribution function. We will then use the derived threshold to calculate the conceptual intervention group probability (*p*_*E*_). The intervention group mean response is assumed to follow a normal distribution with mean SMD and a SD of 1. Thus, the intervention group probability is *p*_*E*_ = 1 − *Φ*(*T* − *SMD*). Having estimated the conceptual meta-analysis control and intervention group probabilities from the pooled SMD, we will calculate the OR as follows:

$$\mathrm{OR}=\frac{p_{E}\left(1-p_{C}\right)}{p_{C}\left(1-p_{E}\right)}$$

To calculate the 95% CI, we will use the above formulas substituting the upper and lower bounds of the SMD confidence interval. We will complement this presentation by converting the SMD into natural units of a widely accepted instrument used to measure changes in the domain of interest or, if such an instrument is not available, we will calculate the ratio of means [58].

### Assessment of heterogeneity and subgroup analyses

We will examine heterogeneity of meta-analyses using both a chi-squared test and the I^2^ statistic, the percentage of among-study variability that is due to true differences between studies (heterogeneity) rather than sampling error (chance) [77,78]. We have generated the following *a priori* hypotheses to explain variability between studies: (1) interventions will show larger effects among trials enrolling ≥50% patients clearly defined as meeting the American College of Rheumatology (ACR) criteria for fibromyalgia [1] versus trials in which less than 50% of subjects meet the ACR criteria; (2) larger effects in trials of patients in which receipt of disability benefits or involvement in litigation was an exclusion criteria versus those that did not exclude patients on this basis; and (3) studies with greater risk of bias will have larger effects than studies with lower risk of bias. We will perform subgroup analyses on a component-by-component basis if we detect variability within the individual risk of bias components. We will conduct z-tests [79] to establish if subgroups differ significantly from each other [80].

### Multiple comparison meta-analysis

We will examine the assumptions of similarity (for an indirect treatment comparison) and consistency (for mixed-treatment comparison) before conducting a network meta-analysis. To assess relative effects of competing treatments, we will construct a random effects model within the Bayesian framework using Markov chain Monte Carlo methods in WinBUGS (MRC Biostatistics Unit, Cambridge, UK) [81]. We will model outcomes in every treatment group of every study and specify the relations among the effect sizes across studies [82]. This method combines direct and indirect evidence for any given pair of treatments. We will use the resulting 95% credible intervals (CrIs) to assess treatment effects [83]. We will assess the probability that each treatment was the most efficacious regimen, the second best, the third best, etcetera, by calculating the effect size for each treatment compared with an arbitrary common control group and counting the proportion of iterations of the Markov chain in which each treatment has the highest effect size, the second highest, etcetera. A key assumption behind multiple treatments meta-analysis is that the analyzed network is consistent; that is, that direct and indirect evidence on the same comparisons do not disagree beyond chance. We will locate and estimate inconsistencies by employing a mixed treatment comparisons inconsistency model in the Bayesian framework [84].

We will use a recent user’s guide published in the Journal of American Medical Association (of which two of our team were authors: KT and GHG) to assess the strength of inferences and credibility of our network meta-analysis [85]. We will use this guide to critically appraise our work in 3 domains and 12 subdomains (Table 1).

**Table 1:** **Critical appraisal guide for a Network Meta-Analysis**[85]

***Critical appraisal criteria***	***Specific items***
**A. Are the results of the study valid?**	
	Did the review explicitly address a sensible clinical question?
	Was the search for relevant studies exhaustive?
	Were there major biases in the primary studies?
**B. What are the results?**	
	What was the amount of evidence in the network?
	Were the results similar from study to study?
	Were the results consistent in direct and indirect comparisons?
	What were the overall treatment effects and their uncertainty, and how did the treatments rank?
	Were the results robust to sensitivity assumptions and potential biases?
**C. How can I apply the results to patient care?**	
	Were all patient-important outcomes considered?
	Were all potential treatment options considered?
	Are any postulated subgroup effects credible?
	What is the overall quality and what are limitations of the evidence?

### Confidence in pooled estimates of effect

Reviewers will, independently and in duplicate, assess the confidence in effect estimates for all outcomes using the GRADE (Grading of Recommendations, Assessment, Development and Evaluation) rating system [86]. In the GRADE system of rating quality of evidence for each outcome, randomized trials begin as high quality evidence, but may be rated down by one or more of five categories of limitations: (1) risk of bias, (2) consistency, (3) directness, (4) imprecision, and (5) reporting bias [80]. After considering these categories, the confidence in estimates for each outcome will be categorized as follows: ‘high’ quality of evidence (we are very confident that the true effect lies close to that of the estimate of the effect); ‘moderate’ quality of evidence (we are moderately confident in the effect estimate and the true effect is likely to be close to the estimate of the effect, but there is a possibility that it is substantially different); ‘low’ quality of evidence (our confidence in the effect estimate is limited and the true effect may be substantially different from the estimate of the effect); and ‘very low’ quality of evidence (we have very little confidence in the effect estimate and the true effect is likely to be substantially different from the estimate of effect) [86].

We will assess publication bias by visually observing asymmetry of the funnel plot for each outcome. As a rule of thumb [87], tests for funnel plot asymmetry should be used only when there are at least ten studies included in the meta-analysis. Otherwise the power of the tests is too low to distinguish chance from real asymmetry. We will report our results by type of intervention (for example, psychotherapy, analgesics, antidepressants) and focus on patient-important outcomes. We will report all direct comparison data, and only complement these data with indirect comparison data if the strength of inferences from direct comparisons is similar or less than the strength of inferences from indirect comparisons. If there are no direct comparisons available, we will report indirect comparison data only.

### Knowledge translation

We plan to create a stakeholder advisory committee with representation from ambulatory health care providers from across Ontario, Canada as well as from key organizations. We will ensure that we have geographically diverse representation including primary care providers who practice in rural areas of the province. Members of our stakeholder committee will be invited to attend our planning meeting and share their input/advice with members of the review team.

Our team also will engage in an end-of-study knowledge translation workshop. The purpose of this activity will be to share our findings with key relevant stakeholders (researchers, clinicians and decision-makers) in order to identify future opportunities for dissemination, beyond traditional peer-reviewed publications, with our stakeholders, discuss how to maximize uptake of our findings in patient education and clinical practice, and determine future research directions. The overall goal of the workshop is to develop an agenda that will establish directions to develop and implement our research findings into practice.

The following strategies will be used to promote awareness of the stakeholder meeting findings according to the Ottawa Model of Research Use in which information is tailored to specific audiences: (1) distribution of findings to all involved participants for further input, sharing within their organization, and for sharing with their own stakeholders via newsletter, web site, or other methods; (2) presentation at relevant peer-reviewed and community conferences; and (3) publication in an open-source peer-reviewed journal. We anticipate that this meeting will identify new areas of inquiry for research and practice, such as the development of new educational tools for patients and clinicians. We also anticipate that new collaborations and networks will be created that will support the identified work going forward. Any groups identified through the meeting will be included as part of the report back to the stakeholders in order to broadly disseminate the findings.

## Discussion

Our review will evaluate all treatments for fibromyalgia, provide relative effectiveness of treatments, and evaluate the quality of the evidence in a thorough and consistent manner using the GRADE approach [88-90]. Additionally, many existing reviews focus on surrogate outcomes, such as number of tender points, stiffness, range of motion, or laboratory values; we will prioritize patient-important outcomes such as function and quality of life. The results of our systematic review will be of interest to a broad audience including patients diagnosed with fibromyalgia, health professionals managing fibromyalgia, employers, human resource professionals, insurers/compensation boards, and labor groups. Our review will facilitate evidence-based management of patients with fibromyalgia, identify key areas for future research, and provide a framework for conducting large systematic reviews involving indirect comparisons.

## Abbreviations

CrIs: Credible intervals; GRADE: Grading of Recommendations, Assessment, Development and Evaluation; MD: Mean difference; MID: Minimally important difference; OR: Odds ration; RCTs: Randomized controlled trials; SD: Standard deviation; SMD: Standardized mean difference; WMD: Weighted mean difference

## Competing interests

JWB acts as a consultant to Prisma Health Canada, a private incorporated company funded by employers and insurers that consults on and manages long-term disability claims. All other authors report no conflicts of interest.

## Authors’ contributions

JWB, KT and GHG conceived the study design. JWB and SE completed the first draft of the manuscript. All authors reviewed several drafts of the manuscript and approved the final version.

## Supplementary Material

Additional file 1: Appendix A Search Strategy. Click here for file

Additional file 2: Appendix B Data Abstraction Forms. Click here for file
