# Supplementary material for: Systematic review and network meta-analysis of interventions for fibromyalgia: a protocol
Source: Syst Rev. 2013 Mar 13;2:18. doi: 10.1186/2046-4053-2-18 (PMC3610251; doi:10.1186/2046-4053-2-18)
Supplement: Additional file 1: Appendix A — Search Strategy. [file 2046-4053-2-18-S1.doc]

**Appendix A: Search Strategy**

Database: EMBASE <1980 to 2011 Week 07>

--------------------------------------------------------------------------------

1 fibromyalgia.mp.

2 exp FIBROMYALGIA/

3 myofascial pain.mp.

4 exp myofascial pain/

5 fibrositis.mp.

6 exp FIBROSITIS/

7 or/1-6

Database: Ovid MEDLINE(R) <1948 to February week 2 2011>

--------------------------------------------------------------------------------

1 fibromyalgia.mp.

2 myofascial pain syndromes.mp.

3 or/1-2

Database: CINAHL <1982 to February Week 2 2011>

--------------------------------------------------------------------------------

S1 (MH "Fibromyalgia")

S2 TX fibromyalgia

S3 TX fibrositis

S4 (MH "Myofascial Pain Syndromes+")

S5 TX "Myofascial Pain Syndrome*"

S6 TX "myofascial pain"

S7 S1 or S2 or S3 or S4 or S5 or S6

S8 S1 or S2 or S3 or S4 or S5 or S6

Database: AMED (Allied and Complementary Medicine) <1985 to February 2011>

--------------------------------------------------------------------------------

1 exp Myofascial pain syndromes/ or myofascial pain syndromes.mp.

2 fibromyalgia.mp. or exp Fibromyalgia/

3 fibrositis.mp.

4 1 or 2 or 3

Database: Ovid Healthstar <1966 to January 2011>

--------------------------------------------------------------------------------

1 myofascial pain syndromes.mp. or exp Myofascial Pain Syndromes/

2 exp Temporomandibular Joint Dysfunction Syndrome/

3 1 not 2

4 fibromyalgia.mp. or exp Fibromyalgia/

5 3 or 4

Database: PsycINFO <1806 to February Week 4 2011>

--------------------------------------------------------------------------------

1 fibromyalgia.mp. or exp Fibromyalgia/

2 fibrositis.mp.

3 exp Myofascial Pain/ or myofascial pain.mp.

4 1 or 2 or 3

Database: PapersFirst <to March 2 2011>

--------------------------------------------------------------------------------

1 kw: fibromyalgia or (kw: myofascial w pain w syndrome) or kw: fibrositis

2 no limits

Database: ProceedingsFirst <to March 7 2011>

--------------------------------------------------------------------------------

1 kw: fibromyalgia or (kw: myofascial w pain w syndrome) or kw: fibrositis

2 no limits

Database: Cochrane Library <to March 2 2011>

--------------------------------------------------------------------------------

1 (fibromyalgia):ti,ab,kw

2 MeSH descriptor Fibromyalgia, this term only

3 "myofascial pain syndromes":ti,ab,kw

4 "myofascial pain syndrome":ti,ab,kw

5 "myofascial pain":ti,ab,kw

6 MeSH descriptor Myofascial Pain Syndromes, this term only

7 (fibrositis):ti,ab,kw

8 (#1 OR #2 OR #3 OR #4 OR #5 OR #6 OR #7)

Database: Cochrane Database of Systematic Reviews, Database of Abstracts of Reviews of Effects, Central Register of Controlled Trials <to March 7 2011>

--------------------------------------------------------------------------------

1 (fibromyalgia):ti,ab,kw in Cochrane Reviews, Other Reviews and Clinical Trials

2 MeSH descriptor Fibromyalgia, this term only

3 "myofascial pain syndromes":ti,ab,kw in Cochrane Reviews, Other Reviews and Clinical Trials

4 "myofascial pain syndrome":ti,ab,kw in Cochrane Reviews, Other Reviews and Clinical Trials

5 "myofascial pain":ti,ab,kw in Cochrane Reviews, Other Reviews and Clinical Trials

6 MeSH descriptor Myofascial Pain Syndromes, this term only

7 (fibrositis):ti,ab,kw in Cochrane Reviews, Other Reviews and Clinical Trials

8 (#1 OR #2 OR #3 OR #4 OR #5 OR #6 OR #7)
