# Supplementary material for: Systematic review and network meta-analysis of interventions for fibromyalgia: a protocol
Source: Syst Rev. 2013 Mar 13;2:18. doi: 10.1186/2046-4053-2-18 (PMC3610251; doi:10.1186/2046-4053-2-18)
Supplement: Additional file 2: Appendix B — Data Abstraction Forms. [file 2046-4053-2-18-S2.doc]

**Appendix B: Data Abstraction Forms**

| **FORM I. GENERAL INFORMATION** | | | | | | | | | | | | | | | | | |
| --- | --- | --- | --- | --- | --- | --- | --- | --- | --- | --- | --- | --- | --- | --- | --- | --- | --- |
|  | **Study ID** | |  |  | **Reviewers’ Last name** | - Avrahami | | | | | - Coomes | | | | | | - Malik |
|  |  |  |  |  |  | - Brunarski | | | | | - Connell | | | | | | - Ngo |
|  |  |  |  |  |  | - Bruno | | | | | - Ebrahim | | | | | | - Riva |
|  |  |  |  |  |  | - Burnie | | | | | - Kirmayr | | | | | | - Steenstra |
|  |  |  |  |  |  | - Busse | | | | | - LeBlanc | | | | | | - Torrance |
|  |  |  |  |  |  | - Bala | | | | | - Jankowski | | | | | | - Lesniak |
|  |  |  |  |  |  | - Alexander | | | | | - Faulhaber | | | | | |  |
|  | **Last name of first author** | |  | | |  | **Publication status** | | | | - Peer-reviewed publication - Conference abstract - Other, specify: - Unpublished | | | | | | |
|  | **Full Journal Name** | |  | | |  | **Year** | |  | | |  | **Language** | | | - English - Non-English , specify: ______ | |
|  | **Funding** | | - Exclusively industry-funded - Partially industry-funded | | | | | - No industry funding - Funding unspecified | | | | | | - Not reported - Explicit statement-no funding | | | |
|  | **Conflict of Interest** | | - Yes, one or more of the authors are employees of a company with a vested interest in the trial | | | | | - No conflicts of interest | | | | | | - Not reported | | | |
|  | **Type of report** | | - Main report | | | | | | | - Prolonged follow up | | | | | - Other, specify: . | | |
|  |  |  | - Report of subgroup | | | | | | | - Report of secondary outcome | | | | |  |  |  |
|  | | If this is not main report, please specify the study ID of the main report associated with this study, and the reference citation of the main report | | | | | | | |  | | | | | | | |
|  | | Did the trial start enrolment after July 1, 2005? | | | | | | | | | - Yes | - No | - Not reported | | --- | --- | --- | | | | | | | | |
|  | | If so, is the trial registered? | | | | | | | | | - Yes, provide details:_____ | - No/not reported | | --- | --- | | | | | | | | |

Comment – Form I

|  |
| --- |

| **FORM II. STUDY CHARACTERISTICS** | | | | |
| --- | --- | --- | --- | --- |
|  | **Where was this study conducted**  ***Check all that apply*** | | | - Canada - United States - United Kingdom - Other Europe - Japan - Other Asia - Africa - Australia - New Zealand - South America - Not reported |
|  | **Number of sites** | | | - Single site - 2-5 sites - 6-10 sites - > 10 sites - Not reported |
|  | **Study design** | | | - Parallel trial - Cross-over trial - Factorial trial - N-of-1 trial - Cluster trial |
|  | **Primary outcome(s)** | | | - Specified: ____________________ - Not specified |
|  | **Number of applicable arms** | | - 2 - 3 - 4 - Other, specify | |
|  | **Number of individual approached to take part in the study who chose not to participate** | | | - Reported, specify: ___________ - Not reported |
|  | **Did the trial authors implement a pre-randomization period?** | | | - Yes - No |
|  | **If so, what reason was provided?** | - To establish severity and variability of pain - To identify and exclude patients with high responses to placebo (placebo run-in period) - To identify and exclude patients with intolerable side effects to study treatment (active treatment run-in period) - To treat all patients with the active therapy, and then only randomize responders - To identify and exclude on-adherent patients - Other reason (specify) | | |

| **FORM II. STUDY CHARACTERISTICS - continued** | | |
| --- | --- | --- |
|  | **Was involvement in litigation reported?** | - Yes - No - Unclear |
|  | **If so, was involvement in litigation used as exclusion criteria?** | - Yes - No |
|  | **Was receipt of disability benefits or other wage replacement benefits reported?** | - Yes - No - Unclear |
|  | **If so, was receipt of disability benefits or other wage replacement benefits used as exclusion criteria?** | - Yes - No |
|  | **Duration of treatment (check 1 only)** | - Days:___________ - Weeks: _________ - Months: ________ - Years: __________ |
|  | **Frequency of treatment (check 1 only)** | - Not applicable - Times per day: _________ - Times per week: ________ - Times per month: __________ |
|  | **Duration of the individual treatment unit (check 1 only)** | - Not applicable - Minutes: _________ - Hours: ________ - Other, specify: __________ |
|  | **Length of follow up from randomization** | - Fixed period → (expand if selected) |
|  |  | - Variable period (*complete all the following as appropriate*)   → Minimum (expand if selected)  → Maximum (expand if selected)  → Median (expand if selected)  → Mean (expand if selected)  → Person years . |

Comment – Form II

|  |
| --- |

| **FORM III. RISK OF BIAS** | | | | | |
| --- | --- | --- | --- | --- | --- |
|  | **How was the randomization sequence generated?** | - Computer generated randomization scheme - Random number table - Tossing coin - Rolling of a dice - Picking allocation from a hat/box - Minimization/dynamic allocation - Other, specify . - Not reported | | | |
|  | **Was allocation adequately concealed?** | - Definitely yes - Probably yes - Probably no - Definitely no | | | |
|  | **How was allocation concealed?** | - Sequentially numbered, opaque, sealed envelope - Sequentially coded medication containers - Central randomization (including telephone, web-based and pharmacy-controlled randomization); - Open random allocation schedule(open-label) - “Concealed”, no method described - Other, specify : _________________ - Not concealed - Not reported | | | |
|  | **Blinding of patients** | - Definitely yes | - Probably yes | - Probably no | - Definitely no |
|  | **Blinding of health Care providers** | - Definitely yes | - Probably yes | - Probably no | - Definitely no |
|  | **Blinding of data collectors** | - Definitely yes | - Probably yes | - Probably no | - Definitely no |
|  | **Blinding of outcome assessors** | - Definitely yes | - Probably yes | - Probably no | - Definitely no |
|  | **Blinding of data analysts** | - Definitely yes | - Probably yes | - Probably no | - Definitely no |
|  | **Study stopped early for benefit** | - Yes | - No | - No clear statement | |
|  | **Study stopped early for harm** | - Yes | - No | - No clear statement | |
|  | **Whether patients were analyzed in the groups to which they were randomized?** | - Yes | - No | - Not reported |  |

| **Method of dealing with LTFU** *(Check all that apply)* | | | | | |
| --- | --- | --- | --- | --- | --- |
|  | Censored at the time of LTFU | - Definitely yes | - Probably yes | - Probably not | - Definitely not |
|  | Complete case analysis | - Definitely yes | - Probably yes | - Probably not | - Definitely not |
|  | Worst case scenario | - Definitely yes | - Probably yes | - Probably not | - Definitely not |
|  | Best case scenario | - Definitely yes | - Probably yes | - Probably not | - Definitely not |
|  | Other sensitivity analysis (specify): | - Definitely yes | - Probably yes | - Probably not | - Definitely not |
|  | None of the LFTU had the outcome | - Definitely yes | - Probably yes | - Probably not | - Definitely not |
|  | All LTFU had the outcome | - Definitely yes | - Probably yes | - Probably not | - Definitely not |
|  | LTFU had same incidence as group | - Definitely yes | - Probably yes | - Probably not | - Definitely not |
|  | LTFU had higher incidence than group | - Definitely yes | - Probably yes | - Probably not | - Definitely not |
|  | Other form of imputation *(specify):* | - Definitely yes | - Probably yes | - Probably not | - Definitely not |
|  | Other *(specify):* | - Definitely yes | - Probably yes | - Probably not | - Definitely not |

| **FORM III. RISK OF BIAS - continued** | | | | | | |
| --- | --- | --- | --- | --- | --- | --- |
|  | **Lost to follow up (LTFU) explicitly reported** | - Explicit statement: LTFU occurred | - Explicit statement: LTFU did not occur | | - No explicit statement about LTFU | |
|  | **LTFU reported separately for each study arm** | - Yes | | - No | | - No explicit statement about LTFU |
|  | **LTFU reported relative to each planned follow-up** | - Yes | | - No | | - N/A *(only one planned)* - N/A (no LTFU) |
|  | **Implications of LTFU discussed** | - Yes | | - No | | - N/A |
|  | **Method of dealing with LTFU explicitly described** | - Yes (open table below) | | - No | | - N/A |

Comment – Form III

|  |
| --- |

| **FORM IV. INTERVENTIONS** | |  |
| --- | --- | --- |
|  | **Treatment Arm 1**  **(check all that apply)** | - **Analgesic: code ___________** |
|  |  | - **Anesthetic: code ___________** |
|  |  | - **Anticonvulsant: code ___________** |
|  |  | - **Anti-Depressant: code ___________** |
|  |  | - **Anti-Emetic: code ___________** |
|  |  | - **Anti-hypertensive: code ___________** |
|  |  | - **Anti-Inflammatory: code ___________** |
|  |  | - **Anti-Viral: code ___________** |
|  |  | - **Bone Growth Stimulant: code ___________** |
|  |  | - **Complementary & Alternative Therapy: code ___________** |
|  |  | - **Dopamine Agonist: code ___________** |
|  |  | - **Exercise: code ___________** |
|  |  | - **Hormone Therapy: code ___________** |
|  |  | - **Immunological Modifier: code ___________** |
|  |  | - **Lifestyle Modification: code ___________** |
|  |  | - **Muscle Relaxant: code ___________** |
|  |  | - **Nutrition & Supplements: code ___________** |
|  |  | - **Psychotherapy: code ___________** |
|  |  | - **Sedative: code ___________** |
|  |  | - **Serotonin Antagonist: code ___________** |
|  |  | - **Stimulant: code ___________** |

| **FORM IV. INTERVENTIONS** | |  |
| --- | --- | --- |
|  | **Treatment Arm 2**  **(check all that apply)** | - **Analgesic: code ___________** |
|  |  | - **Anesthetic: code ___________** |
|  |  | - **Anticonvulsant: code ___________** |
|  |  | - **Anti-Depressant: code ___________** |
|  |  | - **Anti-Emetic: code ___________** |
|  |  | - **Anti-hypertensive: code ___________** |
|  |  | - **Anti-Inflammatory: code ___________** |
|  |  | - **Anti-Viral: code ___________** |
|  |  | - **Bone Growth Stimulant: code ___________** |
|  |  | - **Complementary & Alternative Therapy: code ___________** |
|  |  | - **Dopamine Agonist: code ___________** |
|  |  | - **Exercise: code ___________** |
|  |  | - **Hormone Therapy: code ___________** |
|  |  | - **Immunological Modifier: code ___________** |
|  |  | - **Lifestyle Modification: code ___________** |
|  |  | - **Muscle Relaxant: code ___________** |
|  |  | - **Nutrition & Supplements: code ___________** |
|  |  | - **Psychotherapy: code ___________** |
|  |  | - **Sedative: code ___________** |
|  |  | - **Serotonin Antagonist: code ___________** |
|  |  | - **Stimulant: code ___________** |

| **FORM IV. INTERVENTIONS** | |  |
| --- | --- | --- |
|  | **Treatment Arm 3**  **(check all that apply)** | - **Analgesic: code ___________** |
|  |  | - **Anesthetic: code ___________** |
|  |  | - **Anticonvulsant: code ___________** |
|  |  | - **Anti-Depressant: code ___________** |
|  |  | - **Anti-Emetic: code ___________** |
|  |  | - **Anti-hypertensive: code ___________** |
|  |  | - **Anti-Inflammatory: code ___________** |
|  |  | - **Anti-Viral: code ___________** |
|  |  | - **Bone Growth Stimulant: code ___________** |
|  |  | - **Complementary & Alternative Therapy: code ___________** |
|  |  | - **Dopamine Agonist: code ___________** |
|  |  | - **Exercise: code ___________** |
|  |  | - **Hormone Therapy: code ___________** |
|  |  | - **Immunological Modifier: code ___________** |
|  |  | - **Lifestyle Modification: code ___________** |
|  |  | - **Muscle Relaxant: code ___________** |
|  |  | - **Nutrition & Supplements: code ___________** |
|  |  | - **Psychotherapy: code ___________** |
|  |  | - **Sedative: code ___________** |
|  |  | - **Serotonin Antagonist: code ___________** |
|  |  | - **Stimulant: code ___________** |

| **FORM IV. INTERVENTIONS** | |  |
| --- | --- | --- |
|  | **Control Arm**  **(check all that apply)** | - **Analgesic: code ___________** |
|  |  | - **Anesthetic: code ___________** |
|  |  | - **Anticonvulsant: code ___________** |
|  |  | - **Anti-Depressant: code ___________** |
|  |  | - **Anti-Emetic: code ___________** |
|  |  | - **Anti-hypertensive: code ___________** |
|  |  | - **Anti-Inflammatory: code ___________** |
|  |  | - **Anti-Viral: code ___________** |
|  |  | - **Bone Growth Stimulant: code ___________** |
|  |  | - **Complementary & Alternative Therapy: code ___________** |
|  |  | - **Dopamine Agonist: code ___________** |
|  |  | - **Exercise: code ___________** |
|  |  | - **Hormone Therapy: code ___________** |
|  |  | - **Immunological Modifier: code ___________** |
|  |  | - **Lifestyle Modification: code ___________** |
|  |  | - **Muscle Relaxant: code ___________** |
|  |  | - **Nutrition & Supplements: code ___________** |
|  |  | - **Placebo** |
|  |  | - **Psychotherapy: code ___________** |
|  |  | - **Sedative: code ___________** |
|  |  | - **Serotonin Antagonist: code ___________** |
|  |  | - **Stimulant: code ___________** |
|  |  | - **Waiting List** |

Comment – Form IV

|  |
| --- |

| **Form V: PATIENT CHARACTERISTICS** | | | | | | | |
| --- | --- | --- | --- | --- | --- | --- | --- |
|  | **What clinical conditions were studied?** | | - Fibromyalgia - Generalized myofascial pain syndrome - Fibrositis - Muscular rheumatism - Chronic, generalized pain syndrome | | | | |
|  | **Were explicit criteria used to identify participants?** | | - Unequivocal clear and explicit criteria - Some criteria, but not as clear or explicit as desirable - Uncertain - Not reported - Reported in a prior publication | | | | |
|  | **Did ≥50% of participants clearly meet the diagnostic criteria for fibromyalgia according to the American College of Rheumatology [ACR] criteria, 1990?** | | - Yes - No - Uncertain | | | | |
|  | | **Measure** | **Tx Group 1** | **Tx Group 2** | **Tx Group 3** | **Control Arm** | **Total** |
|  | **Duration of chronic pain condition before randomization (in years)**   - Not reported | Mean, SD | , . | , . | , . | , . | , . |
|  |  | Median, IQR | , . | , . | , . | , . | , . |
|  | **Age (year)**   - Not reported | Mean, SD | , . | , . | , . | , . | , . |
|  |  | Median, IQR | , . | , . | , . | , . | , . |
|  | **Number of female participants**   - Not reported | Raw number |  |  |  |  |  |
|  | **Involved in litigation**   - Not reported | Raw number |  |  |  |  |  |
|  | **Receiving disability or other wage replacement benefits**   - Not reported | Raw number |  |  |  |  |  |
|  | **Intensity of required participation** |  | - High - Low | - High - Low | - High - Low | - High - Low | - High - Low |
|  | **Compliance with treatment**   - Not reported | Percentage |  |  |  |  |  |

Comment – Form V

|  |
| --- |

**FORM VI: PARTICIPANT FLOW THROUGH STUDY**

|  | | **Tx Group 1** | **Tx Group 2** | **Tx Group 3** | **Control arm** | **Total** |
| --- | --- | --- | --- | --- | --- | --- |
|  | **Patients randomized (raw number)**   - **Not reported** |  |  |  |  |  |
|  | **Patients mistakenly randomized, appropriately excluded (raw number)**   - **Not reported** |  |  |  |  |  |
|  | **Patients mistakenly randomized, inappropriately excluded (raw number)**   - **Not reported** |  |  |  |  |  |
|  | **Lost to follow-up: withdrew consent (raw number)**   - **Not reported** |  |  |  |  |  |
|  | **Lost to follow-up: withdrew due to adverse effects (raw number)**   - **Not reported** |  |  |  |  |  |
|  | **Lost to follow-up: withdrew due to lack of improvement (raw number)**   - **Not reported** |  |  |  |  |  |
|  | **Lost to follow-up: withdrew due to loss of contact or migration (raw number)**   - **Not reported** |  |  |  |  |  |
|  | **Lost to follow-up: withdrew due to Other Reasons (raw number)**   - **Not reported** |  |  |  |  |  |

**FORM VII: OUTCOMES**

| 1. **Binary Outcome**  - **Reported** - **Not reported → skip this table** | **Number of events / total (denominator)** | | | | | | | | | | | | | |
| --- | --- | --- | --- | --- | --- | --- | --- | --- | --- | --- | --- | --- | --- | --- |
|  | **Tx Group 1** | | | **Tx Group 2** | | | | **Tx Group 3** | | | | **Control Arm** | | |
|  | **Events** | **Total** | Person-years | **Events** | | **Total** | Person-years | **Events** | **Total** | | Person-years | **Events** | **Total** | Person-years |
| **Total** |  |  |  |  | |  |  |  |  | |  |  |  |  |
| **Outcome Code** |  | | | | | | | | | | | | | |
| Is the Endpoint a threshold? | | - **Yes (describe)** | - **No** | | --- | --- | | | | | | | | | | | | | | |
| Is a higher risk better or worse? | | - **Higher risk is better** | - **Higher risk is worse** | | --- | --- | | | | | | | | | | | | | | |
| **Follow-up time** | - **Days: ________** | | | - **Weeks: ________** | | | | - **Months: _______** | | | | - **Years: _________** | | |
| **Effect estimates** | - **Reported** | | | | | | | - **Not reported → skip to next outcome** | | | | | | |
| **Effect measure** | - **RR** | | | - **OR** | | | | - **HR** | | | | - **ARR/RD** | | |
| **Effect estimates** | **Point estimate (95% CI)** | | | | **Unadjusted analyses** | | | | | **Adjusted analyses** | | | | |
|  | **Group 1 vs. Group 2** | | | |  | | | | |  | | | | |
|  | **Group 1 vs. Group 3** | | | |  | | | | |  | | | | |
|  | **Group 1 vs. Control Arm** | | | |  | | | | |  | | | | |
|  | **Group 2 vs. Group 3** | | | |  | | | | |  | | | | |
|  | **Group 2 vs. Control Arm** | | | |  | | | | |  | | | | |
|  | **Group 3 vs. Control Arm** | | | |  | | | | |  | | | | |

| 1. **Continuous Outcome**  - **Reported** - **Not reported → skip this table** | **Score** | | | | | | | | | |
| --- | --- | --- | --- | --- | --- | --- | --- | --- | --- | --- |
|  | **Tx Group 1** | | **Tx Group 2** | | | | **Tx Group 3** | | | **Control Arm** |
|  |  |  |  |  |  |  |  |  |  |  |
| **Unit of measure** | - **Unitless** | | | | | - **Specific unit of measure: ___________________** | | | | |
| **Measure of central tendency** | - **Mean** | | | - **Median** | | | | | - **Mode** | |
| **Measure of variance** |  | |  | | | |  | | |  |
| **Unit of variance measure** | - **SD** | - **SE** | | | - **95% CI** | | | - **IQR** | | - **range** |
| **Outcome Code** |  | | | | | | | | | |
| Is a higher score better or worse? | | - **Higher score is better** | - **Higher score is worse** | | --- | --- | | | | | | | | | | |
| **Follow-up time** | - **Days: ________** | | - **Weeks: ________** | | | | - **Months: _______** | | | - **Years: _________** |
| **Number of patients available for analysis** |  | |  | | | |  | | |  |

**Comment – Form VI**

|  |
| --- |
